# A Multi-Label Detection Deep Learning Model with Attention-Guided Image Enhancement for Retinal Images

**DOI:** 10.3390/mi14030705

**Published:** 2023-03-22

**Authors:** Zhenwei Li, Mengying Xu, Xiaoli Yang, Yanqi Han, Jiawen Wang

**Affiliations:** College of Medical Technology and Engineering, Henan University of Science and Technology, Luoyang 471032, China; 200320221486@stu.haust.edu.cn (M.X.);

**Keywords:** class activation mapping, multi-label classification, data augmentation, model fine-tuning

## Abstract

At present, multi-disease fundus image classification tasks still have the problems of small data volumes, uneven distributions, and low classification accuracy. In order to solve the problem of large data demand of deep learning models, a multi-disease fundus image classification ensemble model based on gradient-weighted class activation mapping (Grad-CAM) is proposed. The model uses VGG19 and ResNet50 as the classification networks. Grad-CAM is a data augmentation module used to obtain a network convolutional layer output activation map. Both the augmented and the original data are used as the input of the model to achieve the classification goal. The data augmentation module can guide the model to learn the feature differences of lesions in the fundus and enhance the robustness of the classification model. Model fine tuning and transfer learning are used to improve the accuracy of multiple classifiers. The proposed method is based on the RFMiD (Retinal Fundus Multi-Disease Image Dataset) dataset, and an ablation experiment was performed. Compared with other methods, the accuracy, precision, and recall of this model are 97%, 92%, and 81%, respectively. The resulting activation graph shows the areas of interest for model classification, making it easier to understand the classification network.

## 1. Introduction

The retina is the light-sensitive layer within the optic nerve tissue on the inner surface of the eyeball. Retinal damage caused by various diseases can eventually lead to irreversible vision loss. With population aging becoming a major demographic trend worldwide, the number of patients with retinal diseases such as age-related macular degeneration (AMD) and diabetic retinopathy (DR) will increase year by year [1,2,3]. Other retinal diseases, including retinal vascular occlusion, hypertensive retinopathy, and retinitis, are important causes of visual impairment. Vision loss can be avoided in most cases if it is diagnosed and treated early in the initial stages. Therefore, more precise screening protocols are needed for the early treatment of high-risk groups to reduce stress on families and the socioeconomic burden of patients with vision loss caused by retinal disease. Screening using fundus images is generally applicable to patients with fundus diseases.

With the improvement of image classification network performance in the field of computer vision [4,5,6], fundus image classification tasks often include the classification of single diseases, such as DR, AMD, and glaucoma disease staging [7,8,9] and multi-disease fundus image classification [10]. Networks commonly used for fundus image classification include Alex Net, VGG Net, ResNet, and EfficientNet. By fusing the training results of multiple models, it can not only learn more features but can also improve the accuracy of the overall model, which is suitable for multi-classification networks. Due to the complexity of fundus diseases, difficulties in the classification of multi-disease fundus images always exist. Firstly, the differences between different fundus images are very slight, and the same fundus lesions are often included in multiple categories. Secondly, the training data are seriously uneven, and some disease datasets are private. Due to the above reasons, it is very difficult to achieve global classification results for multi-disease fundus images.

The number of categories in the RFMiD multi-disease fundus image dataset is 46. For neural networks, the larger the number of categories, the poorer the classification performance [11]. Thus, it is necessary to use the optimization method of neural networks to improve the accuracy and other indicators. For example, by improving activation functions, batching, transfer learning, ensemble learning, and model fusion methods. However, the model fine-tuning technique utilized in transfer learning will ignore diseased areas, which have a major impact on classification outcomes, and this leads to model over-fitting. Diseases with a large patient base and a large amount of public data are diabetic retinopathy, glaucoma, and cataracts, while there is very little data for retinal pigment epithelial changes (RPEC), retinitis choroiditis (CRS), and other diseases. Insufficient model learning results in the problem of high overall classification accuracy but low single-disease classification accuracy. Data augmentation methods amplify the amount of data through transformations. Common methods are folding, rotating, cropping, translation, and adding noise. In the random cropping method, background pixels may be included that are independent of the lesion area, affecting the model’s ability to extract features.

Therefore, the key to multi-disease fundus image classification is how to improve the classification accuracy of each disease when the dataset is unevenly distributed, and its amount is small. In view of this, this paper proposes an integrated network multi-disease classification model based on Grad-CAM [12,13,14] data enhancement to improve classification accuracy on uneven datasets. The gradient-weighted class activation mapping (Grad-CAM) generated by a convolution neural network is used as the data enhancement module.

## 2. Related Work

### 2.1. Fundus Image Classification

Multi-layer convolution kernels are used to extract image features such as color and texture, which are shallow features, while deep features include more abstract aspects when utilizing deep learning for fundus image classification tasks. Better extraction and identification of these features is the key to improving classification network performance.

Attention modules are commonly added to the network to help the model pay more attention to the lesion area on the fundus image. According to studies on the attention module, they can be broadly split into space-level and channel-level attention mechanisms [15], which have applications in various tasks, such as image classification and segmentation [16,17]. Xi Xu et al. [18] utilized the channel attention mechanism in combination with the maximum mean difference to extract fundus image features from glaucoma patients, which can flexibly adjust the input data to focus on the key areas for glaucoma classification.

Liu et al. [19] designed attention-based convolutional neural networks (CNNs) for glaucoma detection, which, unlike other attention-based CNN methods, are also visualized as local lesion areas to improve the performance of glaucoma detection. Lin et al. [20] fused input images and lesion information using attention-based mechanisms to identify diabetic retinopathy. The detection model can learn the weights between the original image and the lesion information, reducing the impact of missing annotations. Jun et al. [21] proposed a fine-grained image classification based on attention-induced image enhancement, which knows the image enhancement process through attention maps and studies the impact of image enhancement on the classification network. Tao et al. [22] used an attention map as a guide and cropped and down-sampled the images to reduce the background noises introduced in the process. Guo Wenming et al. [23] used a class activation map to enlarge and crop the image attention area, which guided the model to learn more subtle feature differences and improve the model’s feature extraction ability.

In addition to adding modules to the network, other deep learning techniques have also made great progress. For example, dropout can reduce the risk of overfitting by introducing regularization. The rectified linear unit (ReLU) solves the problem of gradient disappearance or explosion to some extent, making deeper networks easier to train. Batch normalization (BN) speeds up the network training process. Global average pooling (GAP) significantly reduces the total training parameters [24,25] and effectively reduces the risk of overfitting. J. He et al. [4] proposed an attention-based feature-weighted fusion network, which extracts the features of both fundus images through ResNet and classifies them after the feature fusion module. The network can classify 8 types of fundus images with an accuracy of 0.934, but a lower kappa value indicates that more samples have been misclassified. Dominik et al. [26] used ensemble learning to combine the prediction results of several heterogeneous deep convolutional neural network models and used cross-validation for data training, which increased the accuracy and reliability of predictions.

Although the existing methods have achieved good results in extracting fundus lesion features [27], the data volume still affects the classification performance of the network, and the classification effect of the network cannot be visually analyzed. Different from the above methods, this paper proposes a data enhancement method guided by Grad-CAM visual attention based on the integrated neural network, which amplifies the fundus image dataset in a targeted manner, helps the model learn rich subtle features, and improves recognition accuracy.

### 2.2. Data Enhancement

In Zalier’s [28] deconvolution method, the accuracy of the classification network is affected by occlusion, rotation, and enlargement of the input images, so basic data augmentation using the above method can improve the network’s performance. Guo Fan et al. [29] used 4854 fundus images in the experimental data, and the dataset was enriched by random contrast, random brightness, random gamma transform, random saturation, random cropping, random rotation, and horizontal flipping to increase sample diversity. Wu Xue et al. [30] used translation, flip, and rotation methods to enhance the data of positive samples and compared the data to enhance the network’s performance before and after. It was found that the data-enhanced network can gradually restrain, reducing the risk of overfitting. Tan Run et al. [31] used semantic information to cut the original image to achieve data enhancement, and the enhanced semantic type of image paid more attention to the local detail information of the classification target to further improve the classification accuracy. Xu et al. [32] proposed a local attention network to process the cataract classification task, which improved the performance of cataract classification by acquiring cataract identification features such as the optic disc and the vascular region through local attention.

## 3. Methodology

**Figure 1:**
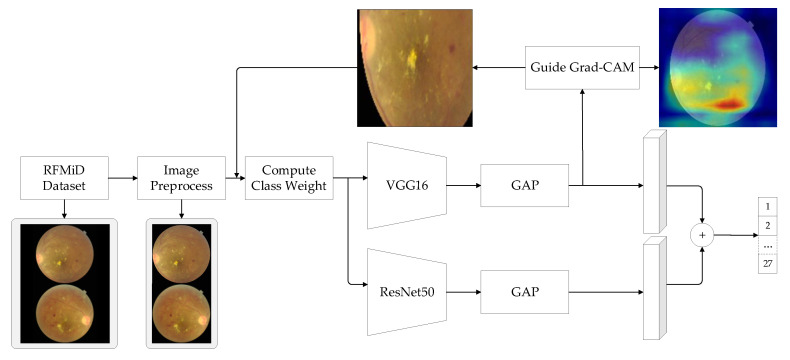
Model structure.

The model of the multi-label classification method is shown in Figure 1. The training set is input to the convolutional network to extract features to obtain the feature map, and the Grad-CAM map is constructed using the feature map and the real label. Using the attention mechanism of the Grad-CAM graph, the original image is cropped to generate different training images, which are input into two convolutional networks for training. Finally, the outputs of the two networks are fused to obtain the final classification result.

The most advanced medical image classification technique is the deep convolutional neural network model. In it, the hyperparameter setting and the choice of model structure highly affect the results of the computer vision task. Therefore, the model is a classifier for multi-label labeling of abnormal images. The model shown in Figure 1 combines two different types of CNN networks, VGG16 and ResNet50, and is represented as BaseModel1 and BaseModel2, respectively.

### 3.1. Data Enhancement

The earliest visualization method used was to introduce deconvolution into the original network to visualize the feature map. However, due to the need to change the network structure and large amounts of computation, class activation mapping was introduced as a new classification network visualization method. In the literature [33], it was proposed that each layer of a convolutional neural network will provide the location information of the target, but it disappears after passing through the fully connected layer. Using global average pooling (GAP) instead of the fully connected layer not only reduces the number of parameters but also preserves location information. Guided backpropagation in combination with gradient-weighted class activation mapping is used to produce high-resolution detail.

Grad-CAM [10] is a general form of CAM that can be applied to any deep learning model with a convolutional structure. Usually, the last convolutional layer can be selected to calculate Grad-CAM. Suppose the output mapping of the last convolutional layer is denoted as $A^{k}$, where k is the number of these output maps. The final Grad-CAM can be calculated as follows:

(1) $$w_{k}^{c}=\frac{1}{Z}\sum_{i=1}^{W}\sum_{j=1}^{H}\frac{\partial y^{c}}{\partial A_{ij}^{k}}$$

(2) $$I_{Grad-CAM}^{c}=ReLU(\sum_{k=1}^{K}w_{k}^{c}\cdot A^{k})$$

where $y^{c}$ represents the scores of class c before the softmax layer. The size of $A^{k}$ is W×H. Pass $y^{c}$ to each $A^{k}$ of differential operations, and $w_{k}^{c}$ is obtained because the class *c* and Z mapping $A^{k}$ are weighted as a normalization factor. In mapping $A^{k}$ after the weighted summation, the activation function of the linear modified unit (ReLu) is applied.

In addition, by modifying ReLu gradient backpropagation, the fraction less than 0 is not propagated, and only the fraction higher than 0 is propagated. As a result, when the first convolution layer is reached, the gradient acquired is the gradient that is used in further ReLu activation. At this point, we display the gradients and determine which region is important in the network; a Guided Grad-Cam $I_{Guide-Grad-CAM}^{c}$ for each prediction result is calculated by multiplying the backpropagation and the class activation map.

(3) $$I_{Guide-Grad-CAM}^{c}=I_{Guide-Backprop}^{c}\cdot I_{Grad-CAM}^{c}$$

To give the results of the final integrated Guided-Grad-CAM multi-label classification, all of the Guided-Grad-CAMs are combined using normalization.

(4) $$I_{Guide-Grad-CAM}=\frac{1}{Z}\sum_{c=1}^{C}I_{Guide-Grad-CAM}^{c}$$

where *Z* represents the normalization factor and *C* represents the total number of categories classified.

Guided-Grad-CAM captures the most critical attention regions of a category, which were initially applied to CNN visualization and target localization under weakly supervised conditions, and this paper uses it to generate cropped images of attention guidance.

In order to obtain the local area of fundus images with regard to Guided-Grad-CAM, we devised a way to identify the lesion area. Set the masking threshold to θ∈[0,255]; $M_{C}$ represents the image after threshold segmentation:

(5) $$M_{C}=\{\begin{array}{l} 1,I_{Guide-Grad-CAM}>\theta \\ 0,others \end{array}$$

**Figure 2:**
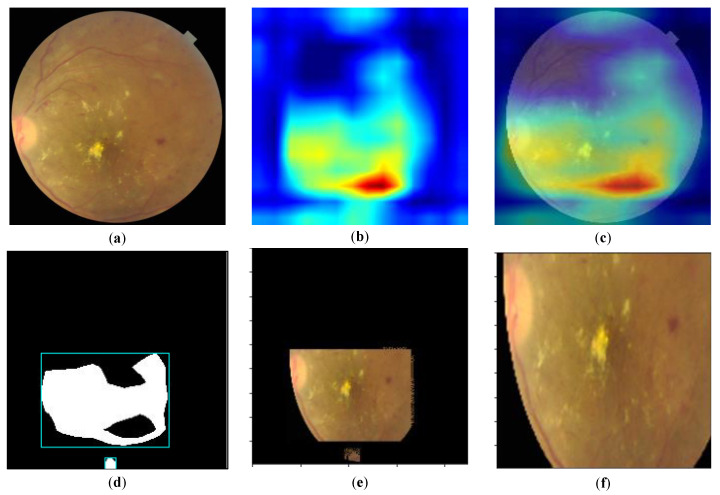
Attention image crop of the (**a**) original image, (**b**) the GradCAM image, and (**c**) the overlay and (**d**) the location of the attention area, (**e**) the attention area clipping, and (**f**) attention area enlargement to the original image size.

Because x,y represent the upper-left coordinates of the smallest circumscribed rectangle of the mask, respectively, h,w represent the height and width of the rectangle, respectively; then, the four-point coordinates of the rectangular area are, respectively, [x,y+h,x+w,y]. As shown in Figure 2, the attention area is obtained by superimposing the mask with the original image, and it is enlarged to the original image size after up-sampling to ensure that it is consistent with the input dimension of the model. Figure 2 shows the process of extracting the image lesion area by the Grad-CAM method. Figure 2a is the fundus image with black edges removed, Figure 2b is the Grad-CAM image of the fundus image, Figure 2c is the superposition of Figure 2a,b, which is used to show the position of Grad-CAM on the original image, Figure 2d is the lesion area cut according to the position of red area in the Grad-CAM image, Figure 2e is the position of the lesion area in the Figure 2a, and Figure 2f is to adjust the length and width of the image in order to input the image into the model.

### 3.2. Feature Fusion

The above enhanced data are fed into the classification networks of BaseModel1 (VGG16) and BaseModel2 (ResNet50). The global average pooling layer is added after the last convolutional layer so that both networks can distinguish the local features of the enhanced data. The two networks are able to extract fundus image features at different depths, which can complement each other to improve predictive performance. The prediction scores of the two networks are combined to obtain the final classification result $G_{f}$:

(6) $$G_{f}=\lambda \times G_{1}+\sigma \times G_{2}$$

where $G_{1}$ and $G_{2}$ indicate the classification results of BaseModel1 and BaseModel2, respectively; λ and σ indicate the weights of each component’s influence (λ+σ=1).

### 3.3. Loss Function Design

Lin et al. [34] used weighted focal loss to make the model more focused on hard-to-classify samples when training by reducing the weight of easily classifiable samples, as follows:

(7) $$FL(p_{c})=-\alpha_{c}{\left(1-p_{c}\right)}^{\gamma }\log(p_{c})$$

where $p_{c}$ is the probability that the class, c is the true value, γ is an adjustable focusing parameter (set to 2.0), and $\alpha_{c}$ is the loss weight of class c.

## 4. Experimental Results and Analysis

In order to verify the effectiveness of the proposed multi-label classification model, this paper performed experiments on the fundus image public dataset. The experimental results from previous studies are compared, and the contributions of the data enhancement algorithm and ensemble model are analyzed. Meanwhile, the classification results are visualized to verify the model’s ability to acquire lesion areas.

### 4.1. Experimental Datasets

**Figure 3:**
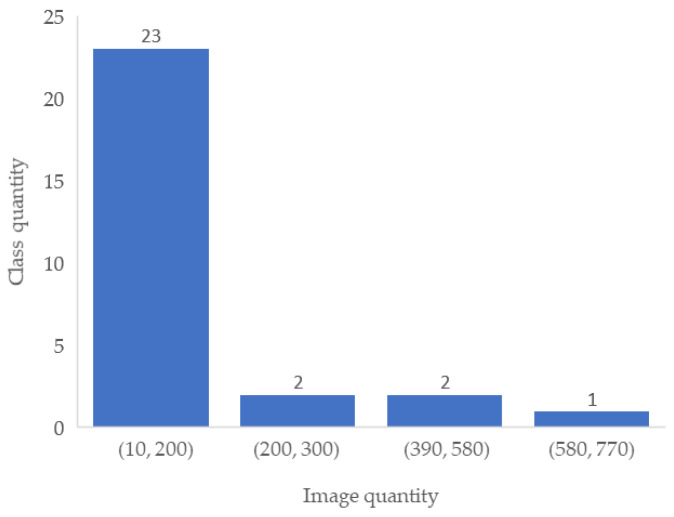
Image quantity histogram distribution.

**Figure 4:**
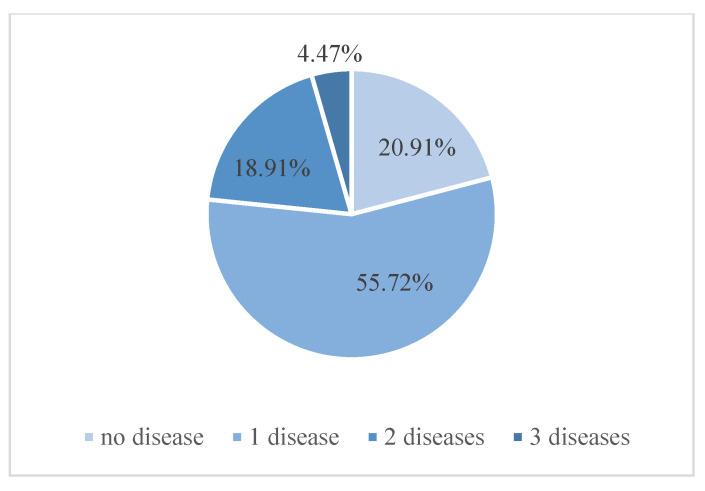
Multi-label image distribution.

The Retinal Fundus Multi-Disease Image Dataset (RFMiD) consists of 3200 images with labels for 45 different diseases. The dataset is divided into 3 subsets: 60% for the training set (1920 images), 20% for the test set (640 images), and 20% for the validation set (640 images). Each subset has 26 diseases labeled independently, and 19 other disease categories are combined and labeled “other”. This ultimately constitutes 28 categories for the classification of diseases. Figure 3 shows the histogram statistics of the number of images versus the number of disease categories in the RFMiD dataset, including the number of images for 23 diseases in the (10, 200) interval and the number of images for only 1 disease in the (580, 770] interval. Figure 4 shows the multi-label image information statistics, and the number of images with only 1 disease in the RFMiD dataset accounts for 55.72%, and the number of images with 2 or more diseases accounts for 23.38%. Table 1 lists the image distribution used for the training set. It can be seen from Figure 4 and Table 1 that the distribution of the image numbers of different categories is uneven, and most images have more than one disease label.

### 4.2. Experimental Parameter Setting

The experiment was based on the Python and Tensorflow deep learning framework and used an RTX 2080Ti GPU to complete accelerated training.

Considering the efficiency and complexity of the network and the cost of training, this study resized all the input images to 224 × 224. The training set was divided into two steps. The VGG16-based framework network was trained on the entire fundus image in the first step, and the local lesion features were extracted and cropped from the original image using Grad-CAM to obtain amplified data. In the second stage, the original dataset and the cropped image were further amplified with random brightness, random gamma transform, random saturation, random cropping, random rotation, and horizontal flipping. Data were fed into the integrated network of VGG19 and ResNet50 for training.

The ImageNet [34] dataset was used to train both VGG19 and ResNet50. Transfer learning training, i.e., frozen architectural layers except for classification heads, and fine-tuning procedures utilizing unfrozen layers, were utilized in the fitting process. The transfer learning fit used Adam to optimize the initial learning rate 1 × 10^4^ and was dynamically lowered to 1 × 10^7^ (reduction factor 0.1) across 10 epochs. Validation set loss increased the learning rate without optimization after eight epochs. Furthermore, for the fine-tuning process, early stop and model checkpointing techniques were used, ending the operation after 20 epochs without improvement and saving the best model evaluated by the verification loss.

The training strategy applied a bagging method based on five-fold cross-validation as ensemble learning, creating different models and training on different subsets of the training data. This approach not only allows for more efficient use of the available training data but also increases the reliability of predictions. This strategy yielded an integration of 10 disease label classifier models (2 structures, each with 5 folds). Finally, the weight parameters that appear in Section 2.2 (λ and σ) were set to (0.6 and 0.4), respectively.

### 4.3. Experimental Results

#### 4.3.1. Classification Performance Evaluation

**Figure 5:**
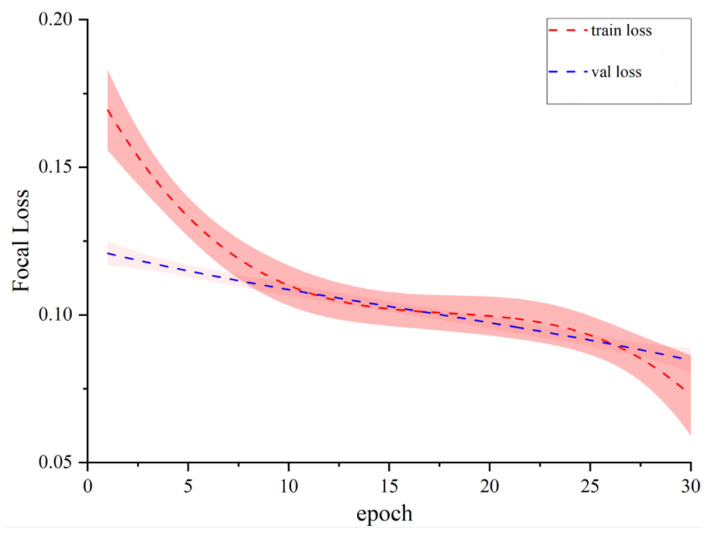
Loss of training set and validation set.

**Figure 6:**
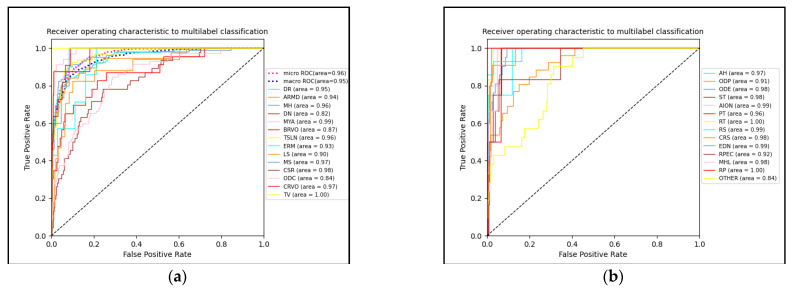
ROC curves of the models: (**a**) ROC curves of some diseases and (**b**) ROC curves of other diseases.

Figure 5 shows the loss function fit of the training and validation sets on the model, with it showing a downward trend. The loss of the validation set gradually exceeds the training set after 26 epochs of data. The lines were computed via locally estimated scatterplot smoothing and represent the average loss across all folds. The red areas around the lines represent the confidence intervals. Figure 6 shows the ROC curve for each disease type, and it can be seen that the ROC curve scores high regardless of the size of the dataset. The average area under the curve is 0.95.

The high class imbalance between the situations indicated a significant problem in developing a reliable model, which is a complicated task in general. Our deployed up-sampling and class weighting approach showed a significant improvement in the classifier models’ prediction abilities. Although the majority of diseases can be correctly classified, the AUC of drusens (DN), optic disc cupping (ODC), and others does not exceed 0.9, which is due to the lack of images for these 3 diseases and their identification characteristics are ambiguous.

**Figure 7:**
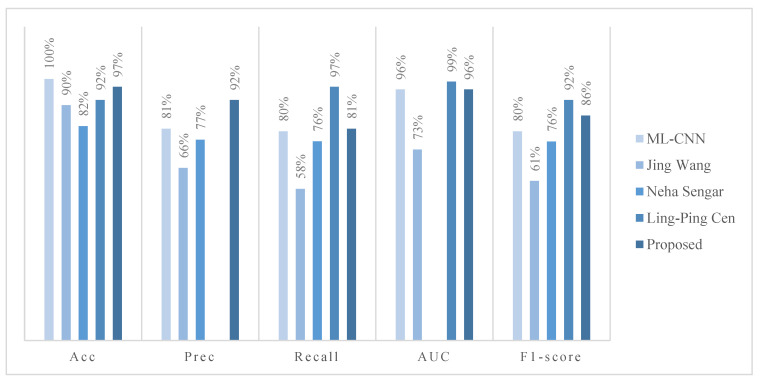
Comparison between some current studies and the proposed model.

Figure 7 provides a detailed analytical comparison of the proposed model with the metrics in other literature sources. ML-CNN [35] reached 100% on Acc and 81% on Prec. Wang et al. [36] achieved an Acc reach of 90%, with Prec and Recall reaching 66% and 58% in the ODIR2019 dataset, respectively. With the exception of Prec and Sen, our model outperforms the model proposed by Wang et al. [36].

Neha Sengar [37] designed an automated deep learning-based non-invasive framework to diagnose multiple eye diseases using an RFMiD dataset called EyeDeep-Net; the accuracy, precision, recall, and F1-score are 82%, 77%, 76%, and 76%, respectively.

Ling-Ping Cen [38] developed a deep learning platform (DLP) capable of detecting multiple common referable fundus diseases and conditions by collecting 3 fundus image datasets, 3 groups of CNNs, and a Mask-RCNN which were applied to construct a 2-level hierarchical system for the classification of the 39 types of diseases and conditions. The accuracy, recall, AUC, and F1-score are 92%, 97%, 99%, and 92%, respectively.

The proposed model has better accuracy, precision, recall, and specificity and a better F1-score than other existing models. The accuracy, precision, recall, AUC, and F1-score are 97%, 92%, 81%, 96%, and 86%, respectively.

#### 4.3.2. Module Comparison Experiment

In order to explore the influence of the above method on the final result, several experiments were performed on the RFMiD dataset. The experimental results are shown in Table 2. Without using any ensemble learning techniques, the accuracy rates obtained using the VGG16 and ResNet50 network models are 90% and 92%, respectively. The method of ensemble learning is used to improve the accuracy of the model. In addition, the model uses the CAM-amplified dataset as the training set to improve accuracy and precision.

#### 4.3.3. Visual Analytics

**Figure 8:**
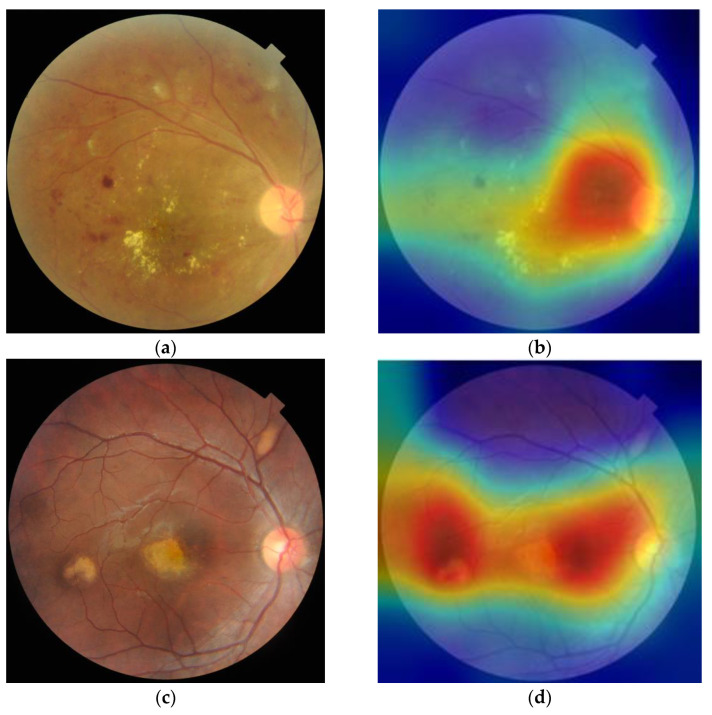

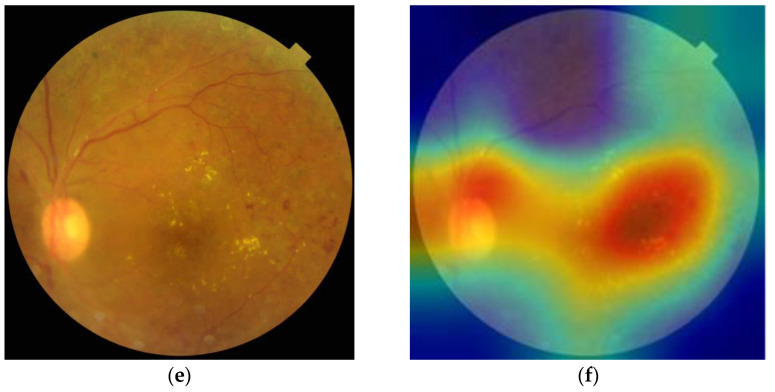
(**a**,**c**,**e**) Original fundus images represents the DR (diabetic retinopathy), CRVO (central retinal vein occlusion), and RPEC (retinal pigment epithelium changes), respectively; (**b**,**d**,**f**) Guided-Grad-CAMs of the multi-label classification model.

Grad-CAM was able to recognize and emphasize the target lesions on the fundus image and used the well-trained multi-label classification model, as shown in Figure 8. It can be seen that for fundus images with lesions, Grad-CAM can locate these areas well and use image cropping to obtain key areas of the image, which can achieve the effect of expanding the dataset.

## 5. Conclusions

In this paper, a multi-label classification model with interpretable Grad-CAM is proposed. Due to the limitations of ophthalmologist resources, simplifying data annotation can greatly increase the amount of valuable data available. In the fundus image labeling stage, this paper developed an attention mechanism for fundus image lesions and performed multi-label classification, which improved the efficiency of labeling work. In order to complete lesion detection on fundus images using the multi-label classification model, Grad-CAM is used to automatically outline each specific lesion area. The experimental results prove the effectiveness and accuracy of this method for disease classification and lesion detection. Furthermore, when fundus images accumulate, deeper lesions or features may be added as independent categories to our multi-label classification algorithm to achieve more accurate lesion locations using Grad-CAM. In the future, more real data will be required to train the network, and DN and ODC’s classification accuracy should be improved. We will investigate the effectiveness of improving loss functions for imbalanced data. Grad CAM may also help with our understanding of the black-box neural network model, and conducting in-depth research into it can help in our understanding of the model’s decision-making process.

## Figures and Tables

**Table 1:** Image quantity of each class in the training set.

Disease Type	Image Quantity	Disease Type	Image Quantity	Disease Type	Image Quantity
DR	376	CSR	37	RS	43
ARMD	100	ODC	282	CRS	32
MH	317	CRVO	28	EDN	15
DN	138	TV	6	RPEC	22
MYA	101	AH	16	MHL	11
BRVO	73	ODP	65	RP	6
TSLN	186	ST	5	Others	34
ERM	14	AION	17	ODE	58
LS	47	PT	11		
MS	15	RT	14		

**Table 2:** Comparative trial.

Model	Accuracy	Precision	Recall	F1 Score
VGG16	0.8969	0.7292	0.7955	0.7609
ResNet50	0.9156	0.7308	0.7451	0.7378
Ensemble model	0.9172	0.7762	0.8409	0.8072
Ensemble model + Image enhance	0.9737	0.9167	0.8083	0.8590

## Data Availability

Data is contained within the article. The data presented in this study are available in [Retinal Fundus Multi-Disease Image Dataset (RFMiD): A Dataset for Multi-Disease Detection Research].

## References

[1] Klein R., Klein B.E.K. (2013). The prevalence of age-related eye diseases and visual impairment in aging: Current estimates. Investig. Ophthalmol. Vis. Sci..

[2] Wong W.L., Su X., Li X., Cheung C.M.G., Klein R., Cheng C.Y., Wong T.Y. (2014). Global prevalence of age-related macular degeneration and disease burden projection for 2020 and 2040: A systematic review and meta-analysis. Lancet Glob. Health.

[3] Li Z., Jia M., Yang X., Xu M. (2021). Blood Vessel Segmentation of Retinal Image Based on Dense-U-Net Network. Micromachines.

[4] He J., Li C., Ye J., Wang S., Qiao Y., Gu L. Classification of Ocular Diseases Employing Attention-Based Unilateral and Bilateral Feature Weighting and Fusion. Proceedings of the 2020 IEEE 17th International Symposium on Biomedical Imaging (ISBI).

[5] Kha Q.H., Tran T.O., Nguyen T.T., Nguyen V.N., Than K., Le N.Q.K. (2022). An interpretable deep learning model for classifying adaptor protein complexes from sequence information. Methods.

[6] Le N.Q.K. (2022). Potential of deep representative learning features to interpret the sequence information in proteomics. Proteomics.

[7] Nneji G.U., Cai J., Deng J., Monday H.N., Hossin M.A., Nahar S. (2022). Identification of Diabetic Retinopathy Using Weighted Fusion Deep Learning Based on Dual-Channel Fundus Scans. Diagnostics.

[8] Pham Q.T., Ahn S., Shin J., Song S.J. (2022). Generating future fundus images for early age-related macular degeneration based on generative adversarial networks. Comput. Methods Programs Biomed..

[9] David D.S., Selvi S.A.M., Sivaprakash S., Raja P.V., Sharma D.K., Dadheech P., Sengan S. (2022). Enhanced Detection of Glaucoma on Ensemble Convolutional Neural Network for Clinical Informatics. Comput. Mater. Contin..

[10] Quellec G. (2020). Automatic Image Analysis Method for Automatically Recognising at Least One Rare Characteristic. U.S. Patent.

[11] Choi J.Y., Yoo T.K., Seo J.G., Kwak J., Um T.T., Rim T.H. (2017). Multi-categorical deep learning neural network to classify retinal images: A pilot study employing small database. PLoS ONE.

[12] Zhou B., Khosla A., Lapedriza A., Oliva A., Torralba A. Learning Deep Features for Discriminative Localization. Proceedings of the 2016 IEEE Conference on Computer Vision and Pattern Recognition (CVPR).

[13] Selvaraju R.R., Cogswell M., Das A., Vedantam R., Parikh D., Batra D. Grad-CAM: Visual Explanations from Deep Networks via Gradient-Based Localization. Proceedings of the IEEE International Conference on Computer Vision.

[14] Chattopadhay A., Sarkar A., Howlader P., Balasubramanian V.N. Grad-CAM++: Improved Visual Explanations for Deep Convolutional Networks. Proceedings of the 2018 IEEE Winter Conference on Applications of Computer Vision (WACV).

[15] Hu J., Shen L., Sun G. Squeeze-and-excitation networks. Proceedings of the CVPR.

[16] Peng Y., Dharssi S., Chen Q., Keenan T.D., Agrón E., Wong W.T., Chew E.Y., Lu Z. (2018). Deepseenet: A deep learning model for automated classification of patient-based age-related macular degeneration severity from color fundus photographs. Ophthalmology.

[17] Zhang Z., Zhang X., Peng C., Xue X., Sun J. Exfuse: Enhancing feature fusion for semantic segmentation. Proceedings of the ECCV.

[18] Xu X., Guan Y., Li J., Ma Z., Zhang L., Li L. (2021). Automatic glaucoma detection based on transfer induced attention network. BioMed Eng. OnLine.

[19] Li L., Xu M., Wang X., Jiang L., Liu H. Attention Based Glaucoma Detection: A Large-Scale Database and CNN Model. Proceedings of the 2019 IEEE/CVF Conference on Computer Vision and Pattern Recognition (CVPR).

[20] Lin Z., Guo R., Wang Y., Wu B., Chen T., Wang W., Chen D.Z., Wu J., Frangi A., Schnabel J., Davatzikos C., Alberola-López C., Fichtinger G. (2018). A Framework for Identifying Diabetic Retinopathy Based on Anti-noise Detection and Attention-Based Fusion. Medical Image Computing and Computer Assisted Intervention—MICCAI 2018. MICCAI 2018.

[21] Lu J., Wu W. (2021). Fine-grained image classification based on attention-guided image enhancement. J. Phys. Conf. Ser..

[22] Hu T., Qi H., Huang Q., Lu Y. See Better before Looking Closer: Weakly Supervised Data Augmentation Network for Fine-Grained Visual Classification. https://arxiv.org/abs/1901.09891.

[23] Guo W., Wang Y. (2021). Class Activation Mapping Guided Data Augmentation for Fine-Grained Visual Classification. J. Comput.-Aided Des. Comput. Graph..

[24] Gao K., Shen H., Liu Y., Zeng L., Hu D. Dense-CAM: Visualize the Gender of Brains with MRI Images. Proceedings of the 2019 International Joint Conference on Neural Networks (IJCNN).

[25] Lin M., Chen Q., Yan S.C. Network in Network. https://arxiv.org/abs/1312.4400.

[26] Müller D., Soto-Rey I., Kramer F. (2021). Multi-Disease Detection in Retinal Imaging based on Ensembling Heterogeneous Deep Learning Models. German Medical Data Sciences 2021: Digital Medicine: Recognize–Understand–Heal.

[27] Li Z., Xu M., Yang X., Han Y. (2022). Multi-Label Fundus Image Classification Using Attention Mechanisms and Feature Fusion. Micromachines.

[28] Zeiler M.D., Fergus R. Visualizing and understanding convolutional networks. Proceedings of the ECCV.

[29] Guo F., Li W., Zhao X., Zou B. (2021). Glaucoma Screening Method Based on Semantic Feature Map Guidance. J. Comput. Des. Comput. Graph..

[30] Wu X., Song X., Gao S. (2020). Convolution Neural Network Based on Data Enhancement for Fire Identification. Sci. Technol. Eng..

[31] Tan R., Tan W., Liu Y. (2022). Fine-Grained Image Classification Combining Dual Semantic Data Augmentation and Target Location. Comput. Eng..

[32] Xu X., Li J., Guan Y., Zhao L., Zhao Q., Zhang L., Li L. (2021). GLA-Net: A global-local attention network for automatic cataract classification. J. Biomed. Inform..

[33] Lin M., Chen Q., Yan S. (2013). Network In Network. arXiv.

[34] Russakovsky O., Deng J., Su H., Krause J., Satheesh S., Ma S., Huang Z., Karpathy A., Khosla A., Bernstein M. (2015). ImageNet Large Scale Visual Recognition Challenge. Int. J. Comput. Vis..

[35] Ouda O., AbdelMaksoud E., El-Aziz A.A.A., Elmogy M. (2022). Multiple Ocular Disease Diagnosis Using Fundus Images Based on Multi-Label Deep Learning Classification. Electronics.

[36] Wang J., Yang L., Huo Z., He W., Luo J. (2020). Multi-Label Classification of Fundus Images With EfficientNet. IEEE Access.

[37] Sengar N., Joshi R.C., Dutta M.K., Burget R. (2023). EyeDeep-Net: A multi-class diagnosis of retinal diseases using deep neural network. Neural Comput. Appl..

[38] Cen L.-P., Ji J., Lin J.-W., Ju S.-T., Lin H.-J., Li T.-P., Wang Y., Yang J.-F., Liu Y.-F., Tan S. (2021). Automatic detection of 39 fundus diseases and conditions in retinal photographs using deep neural networks. Nat. Commun..

